# Retrospective Analysis of 0.25% Bupivacaine for Ultrasound-Guided Infraclavicular and Supraclavicular Nerve Blocks in an Ambulatory Surgery Center

**DOI:** 10.7759/cureus.27391

**Published:** 2022-07-28

**Authors:** Benjamin Rachman, Alec Bigness, Rahul Mhaskar, Nathan Rachman

**Affiliations:** 1 Orthopedic Surgery, University of South Florida Morsani College of Medicine, Tampa, USA; 2 Internal Medicine, University of South Florida Morsani College of Medicine, Tampa, USA; 3 Anesthesiology, Florida State University College of Medicine, Daytona Beach, USA; 4 Anesthesiology, Halifax Health Medical Center, Daytona Beach, USA

**Keywords:** 0.25% bupivacaine, ultrasound guided blocks and vascular access, infraclavicular brachial plexus block, supraclavicular brachial plexus block, ultrasound guided regional anesthesia

## Abstract

Bupivacaine hydrochloride 0.5% (5 mg/mL) is commonly utilized for analgesia in brachial plexus blocks. We suggest that ultrasound-guided 0.25% (2.5 mg/mL) bupivacaine can be utilized for effective postoperative analgesia to reduce the effective dose. A total of 126 patients underwent ultrasound-guided brachial plexus blocks with 0.25% bupivacaine. The mean duration of analgesia was 21.95 (σ = 3.93) hours with no complications. Patients that received an infraclavicular block (22.56 σ = 4.02) had a significant increase in analgesia compared to supraclavicular blocks (21.09, σ = 3.69) (p = 0.04). These results suggest that further research is warranted for ultrasound-guided 0.25% bupivacaine in brachial plexus nerve blocks.

## Introduction

A commonly used anesthetic for postoperative pain relief in patients undergoing upper extremity orthopedic surgery is 0.5% bupivacaine, which is grouped within the amide anesthetics. It is an inexpensive and readily available medication that on average costs around $2.10 per dose [1]. However, a notable downside of bupivacaine for managing postoperative pain from upper extremity surgery is its low therapeutic window and toxicity profile - the most prominent being that of its neurotoxicity and cardiotoxicity [2]. A recent study investigated the formulation of bupivacaine into a liposomal emulsion for enhanced pharmacokinetics, at the risk of complications such as nausea, emesis, and dysgeusia [3]. Its increased price point, however, still casts doubt on its widespread adoption in smaller, ambulatory surgery centers. Advances and recent publications in basic science research aimed at improving drug delivery of bupivacaine show a renewed interest in reducing therapeutic dosages. Nonetheless, judging by the continued interest and research across the community there is a consensus that a smaller therapeutic dose of bupivacaine would be beneficial in managing the risk profile of brachial plexus nerve blocks.

A less documented benefit of bupivacaine use, both in terms of the analgesia's safety and efficacy, is using ultrasound-guided administration. In theory, this technique allows physicians to decrease the dosage concentration from 0.5% to 0.25% with a similar analgesia duration due to enhanced visualization of anesthetic administration around the brachial plexus. The widely used 0.5% bupivacaine has been established as an effective local anesthetic since 1980, if not earlier [4], and has been used long before current technologies like ultrasound were as accessible as they are today. Recent studies show that supraclavicular and infraclavicular blocks of 0.5% bupivacaine without ultrasound range from an average of 11.58 hours [5] to 16.6 hours [6]. A previous study investigated using a 0.25% bupivacaine dose without ultrasound for supraclavicular and infraclavicular nerve blocks, yet only achieved a duration between 9.2 and 13.0 hours [7]. These previous findings are mostly responsible for its perception within the field as inadequate for proper upper-extremity postoperative analgesia. Ultrasound machines have become more accessible and affordable in healthcare and have subsequently increased their use. With more clinicians trained in ultrasound and machines available in clinical sites, the widespread use of ultrasound-guided nerve blocks is easily facilitated. Whether this technology allows for 0.25% bupivacaine to provide a satisfactory duration of analgesia in an outpatient setting while simultaneously increasing the margin of safety for patients has yet to be shown in randomized clinical trials. Altogether, the interest in improving the therapeutic window of bupivacaine combined with recent advances in ultrasound-guided techniques makes the research of this technique against current non-ultrasound-guided practice timely and necessary. We measured the mean duration of analgesia in patients at an ambulatory surgery center to compare from previously reported duration of analgesia.

## Materials and methods

This study evaluated the primary and secondary outcomes of 135 consecutive patients that underwent ultrasound-guided infraclavicular or supraclavicular nerve blocks for upper extremity surgery (excluding any shoulder surgery) at an ambulatory surgery center over a five-month period in 2020. This is a retrospective study utilizing a chart review for the data. The exclusion criteria were age less than 18, any recent history of drug abuse, and/or any rescue nerve block in the post-anesthesia care unit (PACU). One experienced anesthesiologist with over 20 years of clinical practice performed all the nerve blocks for this study.

For each patient, details regarding age, sex, race, American Society of Anesthesiologist (ASA) physical status [8], and diabetes status were collected. Additionally, the duration of the nerve block effect, type of block (supraclavicular or infraclavicular), length of anesthesia onset, and any complications were also collected. We conducted the descriptive analysis to report frequencies for categorical variables and mean and standard deviation for continuous variables. We investigated the correlation between continuous variables by the nonparametric method to calculate Spearman's rho. We investigated the distribution of continuous variables across categorical variables using the Mann-Whitney U test and the Wilcoxson signed-rank test at the significance level of 0.05. We conducted all analyses using IBM SPSS Statistics for Windows, Version 26.0 (Released 2019; IBM Corp., Armonk, United States).

## Results

**Figure 1:**
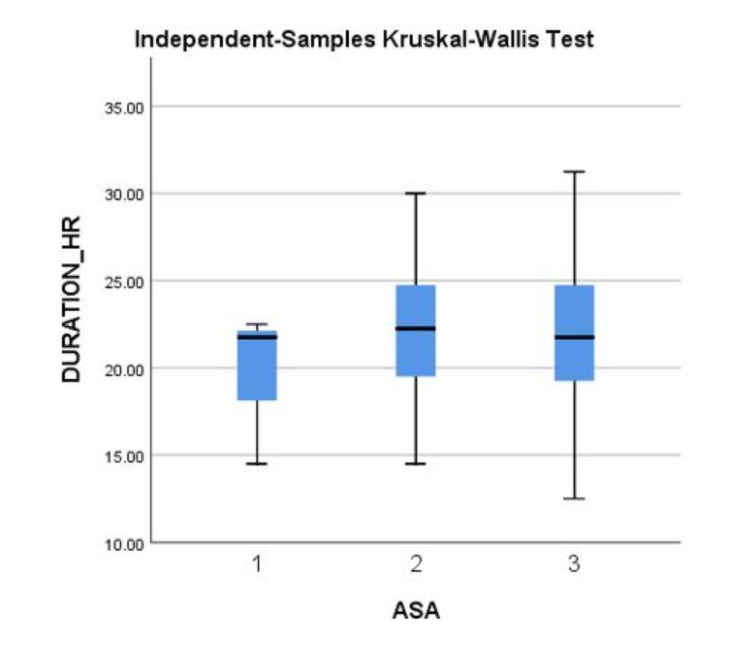
Independent-Samples Kruskal-Wallis test of the ASA score of the patients and their respective duration of analgesia in hours on the Y-axis.

**Figure 2:**
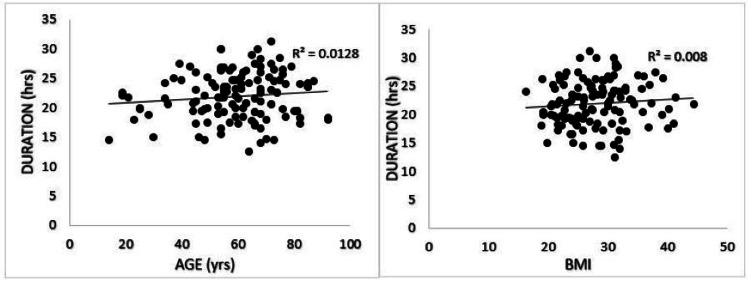
Scatter plots depicting age versus duration of pain relief (left) and BMI versus duration of pain relief (right).

Of the 135 patients considered, 126 were included in the study. No complications were reported. Patient age, ASA score, BMI, and diabetes status were collected with no significant difference between groups. The mean duration of analgesia was 21.95 (σ = 3.93) hours, and there were no significant differences in BMI (p = 0.16, σ = 5.63) on the duration of analgesia. Two categorical age groups were compared with a cutoff of 41 years, and no significant difference in the duration of pain relief was noted between the two groups (p-value = 0.40). Additionally, the ASA score before surgery had no significant impact on analgesia duration for bupivacaine (p-value = 0.63) (Figure 1). It should be noted that diabetic patients had prolonged analgesia from bupivacaine (24.36 hr, σ = 3.93) compared to those without diabetes (21.72 hr, σ = 3.87), (p = 0.01) (Table 1). Also, patients receiving an infraclavicular approach to the nerve plexus block (22.56 σ = 4.02) had prolonged analgesia compared to those that received a supraclavicular approach (21.09, σ = 3.69), (p = 0.04) (Table 2). Continuous variables such as age and BMI are also represented in scatter plots against pain relief duration in Figure 2.

**Table 1:** Comparison of diabetes status and nerve block type with duration of analgesia.

Characteristic	Ultrasound-guided 0.25% bupivacaine (n = 126) duration of analgesia (hours)
Diabetes Status	
Yes (n = 11)	21.72
No (n = 115)	24.36 (p = 0.01)
Block Type	
Infraclavicular (n = 74)	22.56
Supraclavicular (n = 52)	21.09 (p < 0.05)

**Table 2:** Table comparing various characteristics such as sex, diabetes status, and nerve block type on the duration of analgesia from ultrasound-guided 0.25% bupivacaine.

Characteristic	Number of Patients
Sex	
Female	66
Male	60
Diabetes Status	
Yes	11
No	115
BMI	
BMI < 30	82
BMI > 30	40
Age	
Age < 41 yrs	15
Age > 41 yrs	111
Block Type	
Infraclavicular	74
Supraclavicular	52

## Discussion

The present study was effective in demonstrating the use of ultrasound-guided bupivacaine in brachial plexus nerve blocks with a similar duration to other studies without ultrasound. The mean duration of analgesia (21.95 hrs, σ = 3.93) is comparable to the study from Mulroy et al., which showed that femoral nerve blockage with bupivacaine 0.25% and 0.5% provided 23.2 +/- 7 and 25.7 +/- 11 hours of analgesia, respectively [9]. The lack of reported adverse events and a comparable duration of efficacy to femoral nerve blocks without ultrasound guidance, which has been characterized as anatomically easier to access, all point to the safety and efficacy of using ultrasound-guided administration of 0.25% bupivacaine for brachial plexus nerve blocks. Preoperative ASA score was also rejected as a possible confounding factor in the duration of bupivacaine pain relief, as shown in Figure 1. This indicates patients with lower mobility and functionality still benefit equally from this new approach. Additionally, data in Table 1 and Table 2 comparing various preoperative variables such as age and sex show no significant difference between the two groups. Scatter plots (Figure 2) also show no correlation between age or BMI, with the duration of pain relief with ultrasound-guided bupivacaine (R2 of 0.0128 and 0.008, respectively). This data indicates that in comparison to non-ultrasound-guided techniques, bupivacaine 0.25% ultrasound-guided was able to achieve a comparable duration of pain relief without any adverse effects in a cohort of 126 patients.

Diabetics had increased analgesia duration (p = 0.01), which is likely secondary to adverse effects of local anesthetic use on patients with peripheral neuropathy associated with their diabetes have been extensively supported in the literature [10,11]. The destruction of Schwann cells in the peripheral nervous system secondary to sustained hyperglycemia likely plays a role in prolonged pain tolerance [12]. Many diabetic patients have subclinical neuropathy, which may prolong the analgesia from a nerve block [13]. These previous studies and observations likely explain the associated increase in anesthetic duration in patients with diabetes. Our present study adds to the current literature indicating that diabetes status has an associated increased duration of analgesia.

Additionally, it was found that patients with an infraclavicular approach to bupivacaine administration were found to have a significantly longer duration of pain relief (p = 0.04). Previous studies have shown that the infraclavicular route of brachial nerve plexus entry is more effective in reducing pain scores and increasing the length of analgesia when compared to the supraclavicular route [14]. This makes intuitive sense since, traditionally, infraclavicular access is generally easier to achieve. Our results thus substantiate further consensus in the literature.

Our study has multiple limitations. First, it was a single-center study with a single provider. While this limits intraphysician variance, further studies are required to see how the comparison will vary among different anesthesia physicians and to further assess the applicability of this data. Second is a lack of postoperative pain scores, so it is a possibility that patients with longer analgesia duration could have higher pain scores or vice versa. Additionally, a non-ultrasound-guided cohort was not used in the present study for comparative data. Future methods will focus on incorporating a matched non-ultrasound group for comparative measures, and ensure that the results were not due to enhanced user proficiency. Hopefully, these findings will spur more robust studies such as a randomized trial or prospective cohort study to evaluate the use of ultrasound-guided bupivacaine administration against non-ultrasound-guided nerve blocks.

## Conclusions

The study showed that 0.25% ultrasound-guided bupivacaine has similar postoperative analgesia duration to conventional nerve block techniques when combined with ultrasound-guided infraclavicular or supraclavicular nerve blocks, for patients undergoing upper extremity surgery, with no adverse events. Limitations of our study include being a single-center, single-physician study without a matched cohort for comparative data; this impacts the generalizability of the data in other clinical settings. Future studies will include a matched cohort of non-ultrasound-guided administration and address these limitations. However, this study thus prompts further investigation into the use of ultrasound-guided 0.25% bupivacaine brachial plexus nerve block in either a prospective cohort or randomized controlled trial. Overall, these results show promise in possibly reducing the risk and neurotoxicity related to brachial plexus blocks.
